# Development of the Pulmonary Arterial Hypertension-Symptoms and Impact (PAH-SYMPACT®) questionnaire: a new patient-reported outcome instrument for PAH

**DOI:** 10.1186/s12931-016-0388-6

**Published:** 2016-06-14

**Authors:** Deborah McCollister, Shannon Shaffer, David B. Badesch, Arthur Filusch, Elke Hunsche, René Schüler, Ingela Wiklund, Andrew Peacock, IRB information for the 5 clinical sites, Wesley McConnell, Gerald O’Brian, David Badesch, Catherine Markin, Gregory Elliott

**Affiliations:** Division of Pulmonary Sciences and Critical Care Medicine, University of Colorado Denver, 12401 E. 17th Ave., Box L957, Aurora, CO 80045 USA; Outcomes Research, Evidera, 7101 Wisconsin Ave, Suite 1400, Bethesda, MD 20814 USA; Division of Cardiology, University of Colorado Denver, 12401 E. 17th Ave., Box L957, Aurora, CO 80045 USA; Department of Pneumology and Cardiology, HPK – Heidelberg Private Clinic, Im Rossgraben 14, 69123 Heidelberg, Germany; Global Market Access and Pricing, Actelion Pharmaceuticals Ltd, Gewerbestrasse 16, CH-4123 Allschwil, Switzerland; Outcomes Research, Evidera, 1 Butterwick, London, W6 8DL UK; Scottish Pulmonary Vascular Unit, Regional Heart and Lung Centre, Glasgow, G81 4HX UK

**Keywords:** Pulmonary arterial hypertension, Patient-reported outcomes, Health-related quality of life, Symptoms, Activities of daily living

## Abstract

**Background:**

Regulators and clinical experts increasingly recognize the importance of incorporating patient-reported outcomes (PROs) in clinical studies of therapies for pulmonary arterial hypertension (PAH). No PAH-specific instruments have been developed to date in accordance with the 2009 FDA guidance for the development of PROs as endpoints in clinical trials. A qualitative research study was conducted to develop a new instrument assessing PAH symptoms and their impacts following the FDA PRO guidance.

**Methods:**

A cross-sectional study was conducted at 5 centers in the US in symptomatic PAH patients aged 18–80 years. Concept elicitation was based on 5 focus group discussions, after which saturation of emergent concepts was reached. A PRO instrument for PAH symptoms and their impacts was drafted. To assess the appropriateness of items, instructions, response options, and recall periods, 2 rounds of one-on-one cognitive interviews were conducted, with instrument revisions following each round. Additional interviews tested the usability of an electronic version (ePRO). PRO development considered input from an international Steering Committee, and translatability and lexibility assessments.

**Results:**

Focus groups comprised 25 patients (5 per group); 20 additional patients participated in cognitive interviews (10 per round); and 10 participated in usability interviews. Participants had a mean ± SD age of 53.1 ± 15.8 years, were predominantly female (93 %), and were diverse in race/ethnicity, WHO functional class (FC I/II: 56 %, III/IV: 44 %), and PAH etiology (idiopathic: 56 %, familial: 2 %, associated: 42 %). The draft PRO instrument (PAH-SYMPACT®) was found to be clear, comprehensive, and relevant to PAH patients in cognitive interviews. Items were organized in a draft conceptual framework with 16 symptom items in 4 domains (respiratory symptoms, tiredness, cardiovascular symptoms, other symptoms) and 25 impact items in 5 domains (physical activities, daily activities, social impact, cognition, emotional impact). The recall period is the past 24 h for symptoms, and the past 7 days for impacts.

**Conclusions:**

The PAH-SYMPACT® was shown to capture symptoms and their impacts relevant to PAH patients, demonstrating content saturation, concept validity, and ePRO usability. Final content and psychometric validation of the instrument will be based on the results of an ongoing Phase IIIb clinical trial in PAH patients.

**Electronic supplementary material:**

The online version of this article (doi:10.1186/s12931-016-0388-6) contains supplementary material, which is available to authorized users.

## Background

Pulmonary arterial hypertension (PAH) is a rare and debilitating chronic disease of the pulmonary vasculature, characterized by vascular proliferation and remodeling of the small pulmonary arteries [1, 2]. If not treated, it ultimately leads to right heart failure and premature death [3].

The most recent clinical classification scheme for pulmonary hypertension (PH) from the 5th World Symposium on PH distinguishes Group 1 PAH from other forms of PH (Groups 2–5) [4]. Although all PH Groups share symptoms in common [5], there may be differences between types of PH in the relative importance of symptoms and their impacts on patients [6], and potentially also in the types of symptoms.

The most common symptoms reported by patients with PAH are shortness of breath with exertion and fatigue [7]. Symptoms of PAH progress in severity if untreated [3], and may have a major impact on patients’ functioning and physical, psychological, and social well-being [8, 9]; higher rates of depression have been found among PAH patients [10].

Recommendations for PAH trial endpoints from the 4th and 5th World Symposia on PH stress the importance of measuring patient-reported outcomes (PROs) as a secondary endpoint in clinical trials [11, 12]. These developments reflect the rising importance accorded to the patient voice in the drug development and approval process across diseases [13]. Notably, the US Food and Drug Administration (FDA) has implemented a new Patient-Focused Drug Development (PFDD) initiative, intended to bring patient perspectives into early stages of product development [14]. As part of the PFDD, the FDA conducted a public meeting to capture perspectives from patients living with PAH about their disease, its impact on their daily life, and currently available therapies [15].

The FDA has established guidance providing clear scientific standards for clinical outcome assessment [16]. The 2009 FDA guidance for PROs defines a PRO assessment as a report of the status of a patient’s health condition that comes directly from the patient, without interpretation of the patient’s response by a clinician or anyone else [17]. The PRO guidance and the FDA’s 2013 roadmap to patient-focused outcome measurement in clinical trials both emphasize the need to document the content validity of a PRO in patients with the disease [17, 18]. Establishing content validity involves documenting that the structure and content (i.e., items) of the PRO instrument capture the connection between the intended measurement concept and the way patients from the target population understand and discuss that concept [19].

Most PRO questionnaires used in previous PAH clinical trials are generic quality-of-life (QoL) measures that do not adequately reflect the clinical status and symptoms, changes in health status and symptoms, or prognosis of patients with PAH [8, 20]. Other disease-specific PROs that have been used in PAH were either developed in patient populations other than PAH, such as the Minnesota Living with Heart Failure Questionnaire (MLHFQ) [21], or in mixed samples that included PAH patients and patients with other forms of PH. Accordingly, there was a need for a PRO questionnaire developed to assess PAH symptoms and impacts, which can serve as an efficacy endpoint in future PAH studies.

## Methods

### Study design

**Fig. 1:**
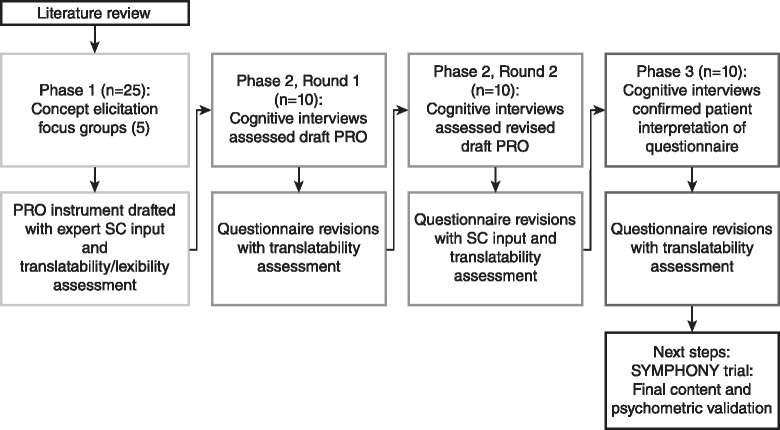
Study flow

In accordance with the FDA roadmap to patient-focused clinical outcome assessment [18], a search was undertaken for existing questionnaires that would meet the PRO guidance requirements for use in PAH studies. No existing PRO measure developed according to the guidance requirements was found, and it was deemed not feasible to modify existing PRO measures. To develop a new disease-specific PRO, a multi-center, cross-sectional, qualitative PRO research study was conducted in patients with PAH in the US between November 3, 2011 and January 9, 2013. The overall study comprised 3 phases (Fig. 1): a concept elicitation phase leading to a draft PRO, a second phase comprising 2 rounds of cognitive interviews, and a final cognitive and usability interview phase based on the electronic version of the instrument (ePRO).

Interviewers used semi-structured interview guides, and interviews were transcribed by a third-party professional transcription service.

The study was conducted in consultation with a Steering Committee (SC) consisting of 3 expert clinicians from the US, the UK, and Germany, and a research instructor with a nursing background from the US. The SC provided clinical expertise and assisted with study design, interpretation of patient responses, item development and item modification, taking patients’ feedback into account. The SC also provided guidance on whether elicited symptoms were generally related to PAH rather than being due to comorbidities or side effects of treatment. The research protocol was approved by the institutional review board of each participating institution. All participants provided written informed consent.

Each phase of the study included a translatability assessment to ensure that the items were appropriate and relevant across cultures, in order to facilitate future translations of the new PRO instrument. Lexibility assessment using the Flesch-Kincaid Grade Level Scale (scored to correspond to school grade level) was conducted after Phases 1 and 3 to ensure that the instructions, items, and response options were easy to understand by individuals with a range of reading levels. A 6th to 9th Grade reading level was considered acceptable, in terms of being inclusive of patients with lower levels of education [22].

#### Phase 1

The concept elicitation phase of the study involved discussions in focus groups, each with 5 PAH patients, using open-ended questions to elicit all concepts regarding PAH symptoms and their impacts on patients’ lives that were considered by patients to be relevant and important. Focus-group discussions were chosen as the appropriate format for concept elicitation as they allow respondents to use the ideas of others as cues to express their own views, and also to compare with their own experiences [19]. In this phase, potential item recall periods and response scale options for the questionnaire were also assessed.

The FDA requires evidence that concept saturation has been reached in the item-generation phase of PRO development [17]. In this context, saturation is defined as the point at which no new relevant or important information emerges and collecting additional data will not likely add to the understanding of how patients perceive the symptoms and impacts of their disease [17]. Accordingly, focus groups in the present study were continued until the point at which no substantially new information or concepts continued to emerge beyond what had been mentioned in previous focus-group discussions [23]. All concepts were summarized in saturation grids for symptoms and for impacts.

Once saturation was reached for symptoms and impacts, a draft PRO questionnaire was developed following generally accepted procedures [24], incorporating feedback from the SC, translatability assessment, and lexibility assessment. The saturation grid was used as the basis for identifying the items to be included in the draft questionnaire. Symptoms due to adverse events were not included, as the objective was to develop a PRO to serve as an efficacy endpoint in future PAH studies. For symptoms of PAH, the threshold for including an item was that at least 20 % of the patients in the total sample (i.e., ≥5 patients) had mentioned it. For impacts, the threshold was emergence of a concept in at least 2 of the 5 focus groups. The rationale for the different thresholds for including symptom vs. impact items was that symptoms, being typically expressed in specific terms (e.g., breathlessness), could be compared across individual patients. In contrast, impacts were often expressed in general terms (e.g., interference with daily activities) and individuals varied in how they described impacts.

For the draft PRO, a draft conceptual framework was developed, which is an organizing tool to depict the relationship of the items to hypothesized domains (i.e., categories of items) [19] for both PAH symptoms and impacts.

#### Phase 2

The overall objective of Phase 2 was to assess the appropriateness of several aspects of the draft PRO instrument, including confirming that PAH patients understood its items as intended and that they considered its items both relevant and comprehensive (i.e., no new concepts emerged in discussions with patients). In addition, Phase 2 assessed the appropriateness of the instructions, response options, and recall periods for symptoms and impacts. The formatting of the draft PRO was also evaluated.

Phase 2 consisted of 2 rounds of semi-structured one-on-one cognitive interviews with different patients from those participating in Phase 1, conducted either face-to-face or by telephone for those patients unable to easily travel to the study centers. Ten patients were included in each round of interviews (10 interviews are generally considered to be sufficient to reach saturation in cognitive interviewing [23]). Inclusion of a second round of cognitive interviews enabled confirmation of revisions to the draft PRO implemented after the first round. Questionnaire revisions followed each interview round, taking into account patient responses, as well as SC input and translatability assessment of any changes.

#### Phase 3

Phase 3 comprised 10 additional face-to-face cognitive interviews (following the same sample-size considerations as for Phase 2) with different patients from those participating in Phases 1 and 2, to confirm the content validity and appropriate patient interpretation of the PRO following limited changes implemented after Phase 2. Phase 3 also included usability testing by the 10 respondents of an electronic version of the questionnaire (ePRO) administered on a tablet computer. Prior to completing the ePRO, participants were provided with instructions and hands-on training on using the tablet. Final revisions of the ePRO followed Phase 3, with continued translatability assessment and lexibility assessment.

### Patient population

Patients meeting study eligibility criteria were recruited from 5 clinical sites in different regions of the US (Table 1). All participants were required to meet the following inclusion criteria: age 18–80 years, inclusive; a definite diagnosis of PAH confirmed by right-heart catheterization; symptomatic in World Health Organization (WHO) Functional Class (FC) I–IV as documented within the past 6 months; and the ability to speak, read, and understand English.

**Table 1:** Study sites

Site number	Phase of study		
	1	2	3
1	X	X	X
2		X	X
3	X	X	X
4		X	
5	X		

To exclude patients with other diseases associated with similar symptoms to those of patients with PAH and/or patients likely unable to participate in interviews, patients meeting the following criteria were not eligible for the study: forms of PH other than PAH; moderate-to-severe obstructive (forced expiratory volume in 1 s/forced vital capacity [FEV_1_/FVC] < 70 %, and FEV_1_ < 65 % of predicted value after bronchodilator administration [25]) or restrictive lung disease (total lung capacity < 60 % of predicted value [26]) at diagnosis; diagnosis of obstructive sleep apnea; any other known concomitant life-threatening disease with a life expectancy <12 months; any other clinically relevant and/or serious chronic medical condition which, in the opinion of the investigator, would interfere with the patient’s ability to participate in an interview; and current participation in a randomized double-blinded clinical research trial that includes the use of investigational medications for any condition. Participants interviewed in one study phase were ineligible for subsequent study phases.

Recruitment aimed to achieve diversity of patients and to be generally representative of the PAH population seen in clinical practice and likely to be recruited in future clinical trials (Table 2). The primary source for developing these recruitment targets was the distribution of patient characteristics at enrolment in the REVEAL registry [27, 28]. Diverse disease severity was targeted to ensure that the PRO would be suitable for use across a wide range of PAH patients. Patients on oxygen therapy were intentionally over-recruited to ensure applicability of the instrument in these patients. Recruitment in Phase 3 aimed to enroll 50 % of patients aged 65 years or older, to ensure that the ability of patients in this age group to use the ePRO could be assessed.

**Table 2:** Patient demographic and clinical characteristics for targeted recruitment and enrolled cohort

	Target	Phase 1: focus groups (*n* = 25)	Phase 2: cognitive interviews (*n* = 20)	Phase 3: cognitive/usability interviews (*n* = 10)	Total sample (*n* = 55)
Age (years)					
<65	Phase 1 & 2: No exact target aside from diversity; Phase 3: 50 %	18 (72 %)	13 (68 %)	8 (80 %)	40 (73 %)
≥65	Phase 1 & 2: No exact target aside from diversity; Phase 3: 50 %	7 (18 %)	6 (32 %)	2 (20 %)	15 (27 %)
Mean ± SD		53.6 ± 15.5	54.3 ± 17.7	49.6 ± 13.1	53.1 ± 15.8
Range		28–76	20–79	28–69	20–79
Gender (n, %)					
Female	75 %–80 %	23 (92 %)	18 (90 %)	10 (100 %)	51 (93 %)
Male	20 %–25 %	2 (8 %)	2 (10 %)	0 (0 %)	4 (7 %)
Ethnicity (n, %)^a^					
Caucasian	70 %–75 %	17 (68 %)	12 (60 %)	8 (80 %)	37 (68 %)
African American	7 %–10 %	4 (16 %)	4 (20 %)	1 (10 %)	9 (16 %)
Hispanic or Latino	7 %–10 %	1 (4 %)	3 (15 %)	2 (20 %)	6 (11 %)
Asian	3 %–5 %	0	0	0	0
American Indian/Alaskan Native		3 (12 %)	0 (0 %)	0 (0 %)	3 (5 %)
Other^b^		0 (0 %)	1 (5 %)	0 (0 %)	1 (2 %)
Highest level of education (n, %)	No exact target aside from diverse educational background				
Elementary/primary school		0 (0 %)	0 (0 %)	1 (10 %)	1 (2 %)
Secondary/high school		8 (32 %)	5 (25 %)	1 (10 %)	14 (25 %)
Some college		12 (48 %)	7 (35 %)	6 (60 %)	25 (45 %)
College degree		2 (8 %)	4 (20 %)	1 (10 %)	7 (13 %)
Postgraduate degree		3 (12 %)	3 (15 %)	1 (10 %)	7 (13 %)
Other		0 (0 %)	1 (5 %)	0 (0 %)	1 (2 %)
Incident vs. prevalent patients					
Newly diagnosed (incident)	10 %–15 %	2 (8 %)	5 (25 %)	2 (20 %)	9 (16 %)
Previously diagnosed (prevalent)	85 %–90 %	23 (92 %)	15 (75 %)	8 (80 %)	46 (84 %)
Patient’s most recent FC					
FC I	5 %–8 %	2 (8 %)	2 (10 %)	0 (0 %)	4 (7 %)
FC II	35 %–40 %	11 (44 %)	8 (40 %)	8 (80 %)	27 (49 %)
FC III	45 %–50 %	11 (44 %)	9 (45 %)	2 (20 %)	22 (40 %)
FC IV	3 %–5 %	1 (4 %)	1 (5 %)	0 (0 %)	2 (4 %)
PAH etiology					
APAH	50 %–55 %	11 (56 %)	9 (45 %)	3 (30 %)	23 (42 %)
IPAH	45 %–50 %	14 (44 %)	10 (50 %)	7 (70 %)	31 (56 %)
FPAH	3 %–5 %	0 (0 %)	1 (5 %)	0 (0 %)	1 (2 %)
Shortness of breath at its worst (numerical rating scale, 0–10), mean ± SD	N/A				
Walking on flat surface		4.0 ± 2.2	4.0 ± 2.9	4.8 ± 3.1	4.1 ± 2.7
Climbing up 1 flight of stairs		6.6 ± 2.5	5.3 ± 3.2^c^	8.1 ± 2.0	6.4 ± 2.9^c^

^a^Not mutually exclusive; ^b^Participant wrote “Jamaican” (*n* = 1); ^c^1 participant did not answer this question (Phase 2 *n* = 19, Total *n* = 54); *APAH* PAH associated with other conditions, *FC* functional class, *FPAH* familial PAH, *IPAH* idiopathic PAH, *N/A* not applicable, *PAH* pulmonary arterial hypertension, *SD* standard deviation

### Assessments

To track patients characteristics vs. the recruitment targets and to characterize patients’ health, assessments administered in each study phase included a participant-completed sociodemographic form, a clinical questionnaire completed by site clinicians, as well as 2 numerical rating scales for shortness-of-breath while walking on a flat surface and climbing up 1 flight of stairs (0–10; 0 = “No shortness of breath at all”; 10 = “Most shortness of breath possible”) and the Medical Outcomes Study 36-item Short Form questionnaire (SF-36, version 2 [29]) which were completed by participants. Participants in Phase 3 also completed a Device Usability Questionnaire assessing the navigation, readability, screen layout, font size, ease of use, ease of selecting an answer, and overall usability of the ePRO.

### Statistical analysis

Content analyses were performed on the qualitative data (i.e., the audio recordings and transcripts) from interviews. Interviews were analyzed using ATLAS.ti v5.0 qualitative data analysis software (ATLAS.ti Scientific Software Development GmbH), to assist with the organization of data and the identification of major concepts. The qualitative data analyses evaluated the saturation of concepts, clarity of instructions, item clarity, interpretation of items, ease of completion, appropriateness of response options and recall periods, and comprehensiveness of the questionnaire (i.e., no missing items).

An item tracking matrix for the PRO was developed to document the item generation and modification process. The item tracking matrix included the rationale for decisions to add, drop, retain, or modify items.

Quantitative data analyses were performed to describe patient demographic and clinical characteristics, as well as the results of the QoL and numeric rating scale assessments. Summary statistics for the quantitative analyses are reported as mean and standard deviation (SD) for continuous variables, and as number and percentage for categorical variables. SF-36 scores were normalized to the adult US population [30, 31].

## Results

### Patient characteristics

A total of 55 patients were enrolled across all 3 study phases: 25 in Phase 1, 20 in Phase 2, and 10 in Phase 3. Patients were diverse in key baseline characteristics (Table 2 and Additional file 1). The overwhelming majority of patients (84 %) were previously diagnosed (i.e., prevalent), and 45 % were using daily oxygen therapy. Nearly all patients (96 %) were taking at least 1 PAH-specific medication, and all were taking some medication to treat PAH (including PAH-specific and nonspecific medications).

**Fig. 2:**
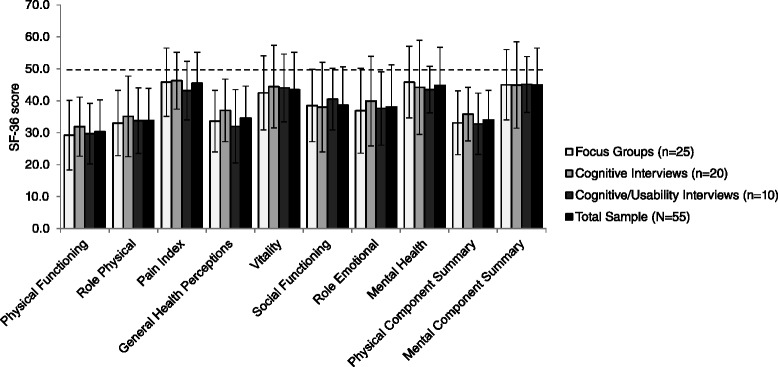
SF-36 results (mean ± standard deviation). Dashed line shows the general population norm score (50) for females and males in the US

Participants had similar SF-36 scores across study phases, which revealed poorer health status in all SF-36 domains in comparison with general population norms in the US, which by definition are 50 for each domain and summary component score (Fig. 2). The greatest health impairments were seen in the domains of physical functioning, role physical, general health, and social functioning. The importance of shortness of breath in PAH was confirmed by results of the numerical rating scales (Table 2).

#### Phase 1

Concept saturation was reached within 5 focus groups for all emergently reported PAH symptoms and within 4 focus groups for impacts (i.e., no previously unmentioned symptoms emerged in the fifth focus group and no new impacts emerged in the fourth and fifth focus groups). Symptoms reported by participants in all 5 focus groups were: shortness of breath, tiredness/fatigue, weakness/lack of energy, swelling (in ankles/legs/hands), rapid heartbeat/heart fluttering, chest pain/tightness, dizziness/lightheadedness, and fainting/passing out. Common impacts reported in at least 4 focus groups included: taking stairs, doing exercise, walking slowly or with difficulty, doing housework, running errands, sleeping difficulties, depression/sadness, frustration/anger, needing help from others, and effects on work or school.

The symptoms and impacts selected for inclusion in the draft PRO are listed in Table 3. To ensure the PRO reflects symptoms and impacts as experienced by patients, care was taken to review the comments actually expressed by the patients in their own words. Examples of comments spontaneously expressed by patients are shown in Table 4.

**Table 3:** Spontaneously reported PAH symptoms and impacts selected for inclusion in the draft PRO

	n (%)
Symptoms (*n* = 25 patients)	
Shortness of breath	23 (92 %)
Tiredness/fatigue/weakness/lack of energy	21 (84 %)
Swelling in ankles or legs	19 (76 %)
Rapid heartbeat/heart fluttering	19 (76 %)
Chest pain/tightness	16 (64 %)
Dizziness/lightheadedness	14 (56 %)
Cough/dry cough	13 (52 %)
Loss of appetite	10 (40 %)
Swelling in stomach area	6 (24 %)
Impacts (*n* = 5 focus groups)^a^	
Physical	
Stairs/exercise	5 (100 %)
Carrying/lifting things	4 (80 %)
Walking slower or with difficulty	3 (60 %)
Activities of Daily Living	
Household chores	5 (100 %)
Errands	5 (100 %)
Work (effects on career or school/reducing hours)	5 (100 %)
Washing/dressing	3 (60 %)
Social Activities	
Needing help from others	4 (80 %)
Going out to social activities	3 (60 %)
Maintaining relationships	3 (60 %)
Feeling embarrassed	2 (40 %)
Cognition	
Concentration/memory/articulating thoughts/slow thinking	3 (60 %)
Emotions	
Sad	4 (80 %)
Frustrated/angry/mad	4 (80 %)
Anxious/worried/stressed/loss of control	3 (60 %)

^a^Refers to difficulties in the listed areas

**Table 4:** Example quotations from participants about their PAH symptoms and impacts

Symptom/Impact	Example quotation
Shortness of breath	I’ve just always had shortness of breath and can hardly breathe sometimes in daily activities …
Tiredness	I’m always tired. …Always. I could just get up from a 4-hour nap, and still want to go back to bed because I’m tired.
Swelling in ankles or legs	… when I used to have it in my legs and my feet, my legs were so heavy that I could hardly even walk, and my feet were so swollen.
Rapid heartbeat	I’ve had my heart rate go up with just sitting. I’m just sitting there and all of a sudden it starts beating real, real fast, and I have to take a few deep breaths to try to get it to slow down.
Physical activities	You know they say walk-how far can you walk. [laughter] Oh, about two blocks and I have to sit down or something, you know.
Activities of daily living	…I’m the cook, and [laughter] used to I’d go in and prepare everything all at one time. Well, now I go in and peel my potatoes and cut them up and put them on the stove or whatever, and I go back and sit down for 20 minutes, and then I go back and prepare something else, until finally I have it all going on the stove cooking, but you have to stretch things out. …
Cognition	Um, yeah, your thinking sometimes just isn’t—how can I—how can I describe it? You’re not able to think things through clearly like you had in the past. Um, there’s a hesitation. You know, you might be asked a question, and there’s a hesitation because you—you’re trying to put the pieces together to respond.
Emotional impacts	I mean, I think the biggest impact is just the worry about it, you know. I worry about getting sicker. I worry about what could happen. You know, I know the stories and how it can go. And you know in the beginning, you read all this stuff, and you think you’re not going to live a year so you start thinking about that.

Based on the saturation grid from the focus-group results and with input from the SC, a draft questionnaire was developed with 16 symptom and 23 impact questions. Activities applicable to both genders were selected for inclusion. Emergent impacts were not included in the draft questionnaire if they were deemed to be infrequently experienced (less than weekly) or unlikely to be shared by most patients (e.g., pregnancy, sexual relations), and would therefore likely lead to missing values.

Similar items potentially measuring the same concepts (e.g., “cough” and “dry cough”) were included in the draft PRO developed after Phase 1; in Phase 2, patients were asked whether these items measure the same concept and if yes, which item should be kept.

A standard 5-point Likert response scale was chosen to assess each item. For symptoms, the response options were graded in terms of severity (no symptom to very severe symptom experience). For impacts, response options were graded either in terms of severity (not at all to very much) or the level of difficulty associated with the performance of the activity (none at all to extreme). Scale selection involved a consideration of both evidence on performance of different response scales described in the literature [32] and participant comments during the interviews: the 0–4 Likert scale with numbers and verbal anchors is commonly used in research practice for rating symptoms in pulmonary disorders and has been found to provide a sufficient range of answer choices to detect changes in symptom severity [33]. The 5-point Likert scale was well received by Phase 1 focus-group participants.

The past 24 h was chosen as an appropriate recall period for symptoms because some patients reported their symptoms changed day-to-day. The past 7 days was chosen as the recall period for impacts because patients may not have the opportunity to carry out impact activities on every day of the week. These recall periods were selected after a review of recall periods that were identified in the literature as being appropriate for assessment of symptoms and impacts of other chronic and debilitating diseases [33–35], and with patient confirmation of their appropriateness.

#### Phase 2

The PRO instrument developed in Phase 1 was named the Pulmonary Arterial Hypertension-Symptoms and Impact (PAH-SYMPACT) Questionnaire®. The draft PAH-SYMPACT® was generally found to be clear, comprehensive, and relevant by PAH patients in the Phase 2 cognitive interviews. No additional concepts beyond those already mentioned in Phase 1 were uncovered in the new sample of patients interviewed in Phase 2.

In the first round of interviews, all participants (*n* = 10, 100 %) reported that the symptom questions applied to their current experiences with PAH and considered the formatting of the questions appropriate. Nearly all participants reported that the impact questions applied to their current experiences (*n* = 9, 90 %). All of the participants that were asked said that the symptom instructions were clear and easy to understand but some (4/9, 44 %) were unsure about whether they should answer impact questions based on whether or not they were using oxygen during the activity. Modifications made to the draft PAH-SYMPACT® after round 1 of the cognitive interviews, following additional input from the translation expert, included:

- A question about oxygen use was added to allow for reporting of symptoms and impacts depending on oxygen use
- One impact question was deleted because participants said it was not applicable
- One impact question was made more specific to avoid variability in item interpretation
- The question stem and response options for several impact items were reworded slightly

No changes were made to the number of symptom items, symptom question phrasing, instructions, or recall period.

In the second round of cognitive interviews using the modified PAH-SYMPACT®, all 10 participants said that the symptom questions applied to their PAH experience, considered the general phrasing of the symptom and impact questions to be appropriate, and confirmed that the instructions were clear and easy to understand. Nine participants (90 %) considered the impact questions applied to their experience. Additional modifications after the round-2 cognitive interviews, made with additional SC and translation-expert input, included:

- One impact question was added and 1 deleted
- Two impact questions were each separated into 2 distinct questions
- Wording of some impact questions was modified slightly

The revised PAH-SYMPACT® after Phase 2 comprised 1 question about oxygen use, 16 symptom questions, and 25 impact questions.

#### Phase 3

In Phase 3, no participant reported difficulty with any feature of the tablet device; all had highly positive overall impressions of using it, and considered it an easy and convenient way to complete the questionnaire.

Based on participant responses in Phase 3, no relevant PAH symptoms or impacts were identified as missing. After patient feedback and input from the translation expert, the following minor modifications were made:

- One symptom item and 1 impact item were reworded slightly
- The example given for 1 impact item was changed to be more relevant
- Some symptom- and impact-item instructions were modified slightly

**Fig. 3:**
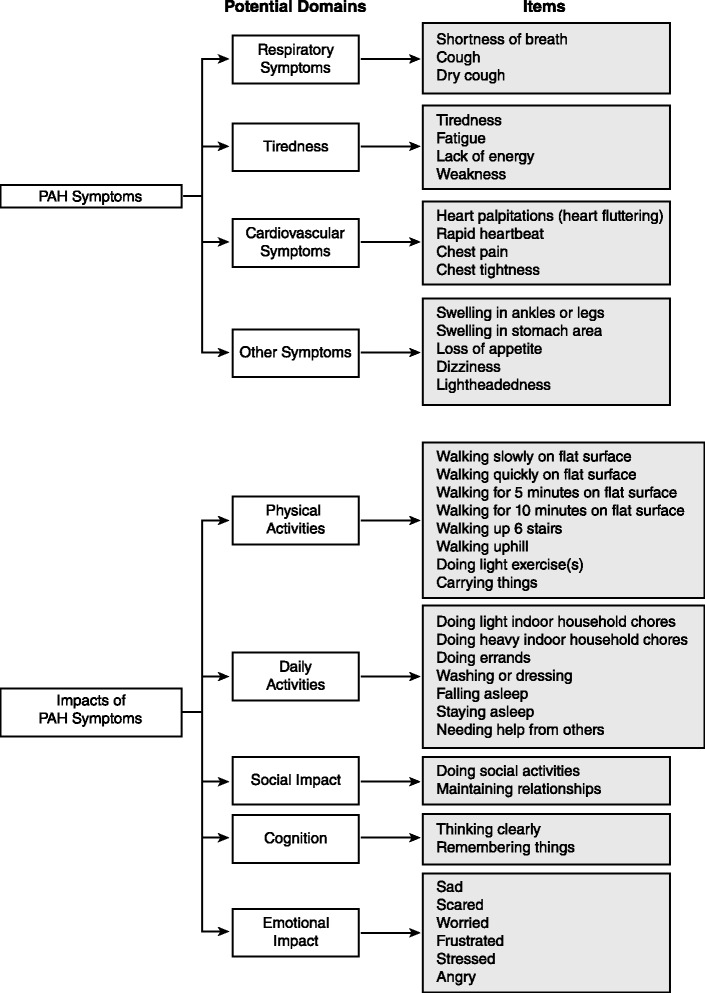
Conceptual framework

The resulting current PAH-SYMPACT® incorporates 4 hypothesized symptom domains comprising 16 items, and 5 hypothesized impact domains comprising 25 items (Fig. 3).

Lexibility analysis indicated that the mean ± SD reading grade level is 1.9 ± 1.7 for symptom items and 3.6 ± 2.8 for impact items. The reason for the higher average reading level for impact items is the need for longer questions to clarify the intended meaning of some impacts; nevertheless, impact items were easily understood by patients. For PAH-SYMPACT® instructions, the reading grade level was 11.0 for symptoms and 12.2 for impacts. The higher scores for instructions are accounted for by the need to identify the disease by its long and complex name, “pulmonary arterial hypertension”. Instructions were easily understood by participants since PAH patients are familiar with the name of their disease and use this term regularly.

## Discussion

The PAH-SYMPACT® questionnaire is the first PRO instrument to be developed for PAH by following strictly the FDA’s PRO guidance. The qualitative research study described here supports the content validity of the PAH-SYMPACT® in a general PAH population in the US, thereby ensuring that the instrument is relevant to patients.

The PAH-SYMPACT® is designed to be a practical tool both for use in general practice and in future trials to assess the effect of PAH and PAH-specific therapies on patients’ symptoms and their impacts. To ensure the potential for its use as an efficacy endpoint in future PAH clinical trials, the PAH-SYMPACT® development process was rigorous with respect to the requirements of the FDA guidance [17]. A stepwise approach was followed, starting with a literature review to confirm the need for a new disease-specific PRO. Patient interviews were conducted in participants representative of both the broader PAH population and the intended population in PAH clinical trials, with recruitment targets being generally met. Patient responses were used as the basis for the development of all questionnaire items, with expert clinician input regarding interpretation of the elicited PAH symptoms and their impacts. The draft PRO was tested with additional PAH patients, whose cognitive interview transcripts demonstrated that no important items were missing and that patients understood the questionnaire’s wording as intended.

Migration of the questionnaire to a tablet device allowing greater convenience in terms of data capture and improved data quality (e.g., avoidance of missing data, automatic date and time stamp) is another strength of the PAH-SYMPACT®. As required by the FDA, a usability study was performed to provide evidence of the feasibility of PAH patients using the ePRO.

The input of a translation expert at each step of questionnaire revision should help to facilitate the adaptation of the PRO to different languages. Lexibility assessment results suggest the PAH-SYMPACT® will be understood even by respondents with relatively basic education. Interview participants were able to correctly restate the instructions in their own words, demonstrating that the questionnaire instructions were well understood.

Previously, only 3 disease-specific PROs have been developed for patients with PH: the Cambridge Pulmonary Hypertension Outcome Review (CAMPHOR) [36], the Living with Pulmonary Hypertension questionnaire (LPH) [37], and the emPHasis-10 questionnaire [38]. However, none of these instruments meet the strict FDA PRO guidance criteria for use in PAH patients. The populations used to develop the CAMPHOR and the emPHasis-10 included patients with some other forms of PH, primarily chronic thromboembolic pulmonary hypertension, and there is no evidence of any discussion of differences of item relevancy or interpretation by PH etiology for either tool [36, 38]. Whether or not the same QoL measure can be used in patients with different forms of PH remains to be determined [20].

In addition, no evidence was reported that a saturation analysis was conducted for CAMPHOR and emPHasis-10 to confirm that all concepts important to patients were captured. Indeed, CAMPHOR is missing concepts such as dizziness, chest pain and palpitations [36], which emerged as important concepts during PAH-SYMPACT® development and which were described by participants in the 2014 FDA PFDD meeting with PAH patients [15]. The emPHasis-10 was developed primarily as a simple scoring system for QoL in PH patients intended for use in clinical practice, not clinical trials, which might explain why the instrument does not cover the full spectrum of PAH symptoms [38].

There was also no demonstration that saturation of symptoms was reached for the LPH, which was an adaptation of an existing measure, the MLHFQ, for use in PAH populations [37]. Although the LPH was developed based on interviews of PAH patients exclusively, the process did not incorporate the potential for addition of new symptoms and impacts beyond those already covered by the MLHFQ. The main modification was a revision to the recall period from 4 weeks to 1 week. Although the latter change was described as an important modification to ensure the instrument met the FDA preference for “short recall periods” [37], a 1-week recall period could introduce recall bias and a daily diary is likely preferable for symptoms, as patients might not be able to average symptom burden over several days.

While the PAH-SYMPACT® development process followed the FDA PRO guidance, there are some considerations when judging its applicability to the intended patient population. No individuals of Asian descent could be enrolled in the qualitative research, despite efforts to meet the recruitment target for this group. Nevertheless, the patient sample may still be considered representative of a clinical-practice population, since the proportion of Asians among US patients with PAH is very low [28]. Based on SC input, no racial differences in symptoms or impacts are anticipated in PAH; thus the lack of Asians in the study population is not expected to diminish the applicability of the questionnaire to this population. Another consideration is that more women were enrolled than seen in clinical practice (93 % vs. 80 % in REVEAL [27]), but since only symptoms and impacts that are generally experienced by both genders were included in the instrument, this should not have impacted the development process. Very few patients with familial PAH (2 %) or in FC IV (4 %) were included, reflecting the difficulty of recruiting patients from these uncommon subgroups.

Although the patients participating in the qualitative interviews were asked about their PAH symptoms and impacts, it may be difficult for patients to attribute symptoms and impacts to their disease. For example, certain PAH-specific therapies are associated with fluid retention or lightheadedness, both of which are also symptoms of PAH. To address this issue, instrument development incorporated feedback from the SC on whether symptoms mentioned by patients were generally PAH-related or potentially side-effects of treatment, guided by analyses of symptoms reported by patients receiving and not receiving different PAH-specific therapies.

## Conclusions

The current PAH-SYMPACT® questionnaire was shown to be a practical and convenient tool for comprehensively capturing PAH symptoms and their impacts relevant to patients. Prior to its use in PAH clinical trials or clinical practice, the PRO needs to undergo final validation steps, including identification of a scoring algorithm, and quantitative testing to determine the psychometric measurement properties of the instrument. This final content and psychometric validation of the PAH-SYMPACT® will be based on the results of the SYMPHONY trial (Study AC-055-401; ClinicalTrials.gov No. NCT01841762), an ongoing Phase IIIb clinical trial in patients with PAH in the US.

## Abbreviations

APAH, PAH associated with other conditions; CAMPHOR, Cambridge Pulmonary Hypertension Outcome Review; EMA, European Medicines Agency; FC, Functional class; FDA, Food and Drug Administration; FPAH, familial pulmonary arterial hypertension; IPAH, Idiopathic PAH; LPH, Living with Pulmonary Hypertension questionnaire; MLHFQ, Minnesota Living with Heart Failure Questionnaire; NYHA, New York Heart Association; PAH, Pulmonary arterial hypertension; PAH-SYMPACT®, Pulmonary Arterial Hypertension-Symptoms and Impact Questionnaire®; PH, Pulmonary hypertension; PRO, Patient-reported outcome; QoL, Quality of life; SC, Steering committee; SD, Standard deviation; SF-36, Medical Outcomes Study 36-item Short Form questionnaire; WHO, World Health Organization

## Additional file

Additional file 1: Table S1. Additional patient demographic and clinical characteristics for targeted recruitment and enrolled cohort. (PDF 34 kb)
