# Supplementary material for: Development of the Pulmonary Arterial Hypertension-Symptoms and Impact (PAH-SYMPACT®) questionnaire: a new patient-reported outcome instrument for PAH
Source: Respir Res. 2016 Jun 14;17:72. doi: 10.1186/s12931-016-0388-6 (PMC4908719; doi:10.1186/s12931-016-0388-6)
Supplement: Additional file 1: Table S1. — Additional patient demographic and clinical characteristics for targeted recruitment and enrolled cohort. (PDF 34 kb) [file 12931_2016_388_MOESM1_ESM.pdf]

Additional file

Supplementary Table. Additional patient demographic and clinical characteristics for targeted recruitment and enrolled cohort

	Target	Phase 1: Focus Groups (n=25)	Phase 2: Cognitive Interviews (n=20)	Phase 3: Cognitive/Usability Interviews (n=10)	Total Sample (n=55)
Current living/domestic situation (n, %)	N/A				
Living with a spouse, partner, or family member		20 (80%)	12 (60%)	9 (90%)	41 (75%)
Living alone		4 (16%)	7 (35%)	1 (10%)	12 (22%)
Living with friends		0 (0%)	1 (5%)	0 (0%)	1 (2%)
Other*		1 (4%)	0 (0%)	0 (0%)	1 (2%)
Employment status (n, %)	N/A				
Not working due to disability		10 (40%)	8 (40%)	4 (40%)	22 (40%)
Full-time paid work		1 (4%)	3 (15%)	2 (20%)	6 (11%)
Retired		6 (24%)	6 (30%)	2 (20%)	14 (25%)
Homemaker		2 (8%)	3 (15%)	1 (10%)	6 (11%)
Part-time paid work		3 (12%)	1 (5%)	1 (10%)	5 (9%)
Student		1 (4%)	0 (0%)	0 (0%)	1 (2%)
Unemployed		2 (8%)	2 (10%)	0 (0%)	4 (7%)
Other[‡]		1 (4%)	0 (0%)	0 (0%)	1 (2%)
Smoking status	N/A				
Not a current smoker		23 (92%)	20 (100%)	9 (90%)	52 (95%)
Current smoker		2 (8%)	0 (0%)	1 (10%)	3 (5%)
Comorbidities (n, %)[†]	N/A				
Hypertension (other than PAH)[‡]		9 (36%)	8 (40%)	2 (20%)	19 (35%)
Obesity (defined as BMI >30)		9 (36%)	5 (25%)	3 (30%)	17 (31%)
Depression[§]		4 (16%)	8 (40%)	1 (10%)	13 (24%)
Congestive heart failure[¶]		9 (36%)	0 (0%)	1 (10%)	10 (18%)
Diabetes[‖]		4 (16%)	2 (10%)	2 (20%)	8 (15%)

	Target	Phase 1: Focus Groups (n=25)	Phase 2: Cognitive Interviews (n=20)	Phase 3: Cognitive/Usability Interviews (n=10)	Total Sample (n=55)
Chronic obstructive pulmonary disease**		1 (4%)	1 (5%)	1 (10%)	3 (5%)
Obstructive sleep apnea		N/A	0 (0%)	3 (30%)	3 (5%)
Kidney disease		1 (4%)	1 (5%)	0 (0%)	2 (4%)
Other††		10 (40%)	7 (35%)	3 (30%)	20 (36%)
None of the above		1 (4%)	4 (20%)	3 (30%)	8 (15%)
Current therapy route of administration					
Parenteral (IV/subcutaneous)	No exact target to meet aside from diversity of therapy route	10 (40%)	8 (40%)	3 (30%)	21 (38%)
Other (oral/inhaled)	No exact target to meet aside from diversity of therapy route	22 (88%)	15 (75%)	10 (100%)	47 (85%)
Current prescribed PAH medication†					
PAH-specific medications					
Endothelin receptor antagonist		14 (56%)	10 (50%)	8 (80%)	32 (58%)
Phosphodiesterase type 5 inhibitor		9 (36%)	13 (65%)	3 (30%)	24 (44%)
Prostacyclin/prostaglandin – parenteral (IV/subcutaneous)		10 (40%)	8 (40%)	3 (30%)	21 (38%)
Prostacyclin/prostaglandin – oral/inhaled		3 (12%)	3 (15%)	0 (0%)	6 (11%)

	Target	Phase 1: Focus Groups (n=25)	Phase 2: Cognitive Interviews (n=20)	Phase 3: Cognitive/Usability Interviews (n=10)	Total Sample (n=55)
Number					
0		2 (3.6%)	0 (0.0%)	0 (0.0%)	2 (3.6%)
1		12 (21.8%)	9 (16.4%)	6 (10.9%)	27 (49.1%)
2		9 (16.4%)	9 (16.4%)	4 (7.3%)	22 (40.0%)
3		2 (3.6%)	2 (3.6%)	0 (0.0%)	4 (7.3%)
Mean ± SD (range)		1.44 ± 0.77 (0–3)	1.65 ± 0.67 (1–3)	1.40 ± 0.51 (1–2)	1.51 ± 0.69 (0-3)
Medications not specific to PAH					
Anticoagulant		9 (36%)	7 (35%)	6 (60%)	22 (40%)
Oxygen therapy[‡‡]		11 (44%)	9 (45%)	5 (50%)	25 (45%)
Using oxygen at least once a day	10%–15%	11 (44%)	9 (45%)	5 (50%)	25 (45%)
Not using oxygen or using oxygen less than once a day	No exact target to meet	14 (56%)	11 (55%)	5 (50%)	30 (55%)
Diuretic		16 (64%)	9 (45%)	4 (40%)	29 (53%)
Calcium channel blocker		11 (44%)	4 (20%)	3 (30%)	18 (33%)
Digoxin/digitalis		1 (4%)	1 (5%)	1 (10%)	3 (5%)
Other[§§]		1 (4%)	1 (5%)	0 (0%)	2 (4%)
Number					
0		2 (3.6%)	2 (3.6%)	3 (5.5%)	7 (12.7%)
1		8 (14.6%)	9 (16.4%)	1 (1.8%)	18 (32.7%)
2		5 (9.1%)	6 (10.9%)	1 (1.8%)	12 (21.8%)
3		9 (16.4%)	2 (3.6%)	4 (7.3%)	15 (27.3%)
4		1 (1.8%)	1 (1.8%)	1 (1.8%)	3 (5.5%)
Mean ± SD (range)		1.96 ± 1.10 (0–4)	1.55 ± 0.10 (0–4)	1.90 ± 1.52 (0–4)	1.80 ± 1.15 (0–4)

*Other "No" (n=1); [‡]Other: "Artist" (n=1); "Diploma RN (3 yrs)" (n=1); [†]Not mutually exclusive; [‡]Hypertension other than PAH: treated (n=2);

[§]Depression: treated (n=11), untreated (n=2); [¶]Congestive heart failure: NYHA I (n=1), NYHA II (n=4), NYHA III (n=3), did not specify (n=1);

[||]Diabetes: treated (n=7), untreated (n=1); **Chronic obstructive pulmonary disease: Gold Stage I (n=2), Gold Stage II (n=1); [††]Other condition:

bipolar affective disorder (n=1), collagen vascular disease-CREST (n=1), idiopathic cardiomyopathy (n=1), atrial septal defect repaired with Amplatzer septal occlude (n=1), asthma (n=1), CREST syndrome (n=2), hypothyroidism (n=4), medical history of transient ischemic attack (n=1), incontinentia pigmenti (n=1), lupus (n=1), rheumatoid arthritis (n=1), monoclonal gammopathy of undetermined significance (n=1), chronic hepatitis (n=1), fatty liver disease (n=1), human immunodeficiency virus (n=1), hepatitis c (n=1), thyroid (n=1), scleroderma (n=1), neurofibrosis (n=1); [‡‡]Oxygen was prescribed for PAH. Hours per day: 24 hours (n=4); 8 hours (n=1); [§§]Other medications: arginine (n=1); BMI: body mass index; CREST: calcinosis, Raynaud's syndrome, esophageal dysmotility, sclerodactyly, telangiectasia; IV: intravenous; N/A: not applicable; NYHA: New York Heart Association; PAH: pulmonary arterial hypertension; SD: standard deviation
